# Molecular Characterization and Gene Expression Analysis of Aquaporin in *Haemaphysalis qinghaiensis*

**DOI:** 10.3389/fphys.2022.811628

**Published:** 2022-02-17

**Authors:** Qingli Niu, Rongzeng Hao, Yuping Pan, Zhijie Liu, Jifei Yang, Guiquan Guan, Jianxun Luo, Hong Yin

**Affiliations:** ^1^State Key Laboratory of Veterinary Etiological Biology, Key Laboratory of Veterinary Parasitology of Gansu Province, Lanzhou Veterinary Research Institute, Chinese Academy of Agricultural Sciences, Lanzhou, China; ^2^Jiangsu Co-innovation Center for Prevention and Control of Important Animal Infectious Diseases and Zoonoses, Yangzhou, China

**Keywords:** ticks, *Haemaphysalis qinghaiensis*, aquaporin, transcript variants, gene expression

## Abstract

Aquaporins (AQPs) are important functional proteins and are widely present in the cell membrane of almost all organisms, mediating transmembrane transport of liquid and other solutes. Much is known about the molecular characterization of AQPs in other tick species; however, nothing is known about them in *Haemaphysalis qinghaiensis*. In this study, we first sequenced the transcript variants of *AQPs* in *H. qinghaiensis* (*HqAQPs*), analyzed the biological structure features of *AQPs*, and investigated the pattern of gene expression of the AQP gene of *H. qinghaiensis* in different tick tissues and stages to predict their biological functions. In conclusion, four *AQP* transcript variants (i.e., *HqAQP1-1*, *HqAQP1-2*, *HqAQP1-3*, and *HqAQP1-4*) of *H. qinghaiensis* were found, and the sequences were comparable with its orthologs from the reported tick species. Gene expression of AQPs in different tick tissues and stages showed the higher expression level in salivary glands and gut of adult female, as well as in the female and nymph than in Malpighian tubules, ovary, male, larvae, and egg. Further studies will be performed to evaluate the function of *HqAQPs* against *H. qinghaiensis* infestation on animals.

## Introduction

Ticks can transmit a wide variety of pathogens to both humans and animals, including viruses, bacteria, and parasites *via* the saliva produced during feeding, and tick saliva is reported to induce Alpha-gal syndrome (Baneth, 2014; Hai et al., 2014; Crispell et al., 2019; Sharma and Karim, 2021); *Haemaphysalis qinghaiensis* belongs to the family of Ixodidae, which was initially identified as a new species by Teng (1980). It is a three-host tick, with a life cycle including egg, larva, nymph, and adult. Larval, nymphal, and adult female ticks can absorb a majority of nutrients from hosts, and this biological trait caused not only a direct impact on the host but also a secondary impact with the potential spread of a variety of pathogens to the host. *H. qinghaiensis* has been proved to be the dominant tick species in the northwest farmland of China (Teng and Zaijie, 1991), is widely distributed in the pastoral areas with an altitude of 1,600–4,200 m in Qinghai, Gansu, Sichuan, Yunnan, and Ningxia provinces, and has not been reported to the rest of the world. It is responsible for the transmission of various pathogens of ruminant livestock, such as *Theileria* spp. and *Babesia* spp., which lead to great harm to the pastoral areas (Guan et al., 2002; Yin et al., 2002a,b,c).

Aquaporins (AQPs) are members of the major intrinsic protein (MIP) superfamily that is widely present in the cell membranes of organisms. They mainly transport water molecules and neutral molecules, such as glycerol, urea, and ammonia, and play a key role in the fluid balance of animal body organs (Reddy and Dony, 2017). In 1988, the Agre group first discovered a 28 kDa hydrophobic membrane intrinsic protein on the red blood cell membrane, which is called the channel-forming integral membrane protein of 28 kDa (CHIP28) (Denker et al., 1988). Its water channel function was then confirmed in the *Xenopus laevis* oocyte expression system (Preston et al., 1992). For this reason, Agre won the Nobel Prize in Chemistry in 2003. Since CHIP28 was the first identified AQP, thus, it was named as aquaporin-1 (AQP1) by the Human Genome Committee.

In piercing and sucking insects, such as *Acyrthosiphon pisum* (Shakesby et al., 2009) and malaria vector mosquitoes, such as *Anopheles gambiae* (Liu et al., 2011) and *Bemisia tabaci* (Mathew et al., 2011), AQPs are involved in regulating the water discharge in the liquid food and also participated in the antifreeze physiology of hardy insects, such as *Antarctic midge* (*Belgica antarctica*) (Goto et al., 2011), *Chilo suppressalis* Walker (*Lepidoptera: Pyralidae*) (Izumi et al., 2006), and the seasonal anti-drying effect of *Callosobruchus maculatus* (*Coleoptera: Bruchidae*) (Yoder et al., 2010). Studies on the abovementioned different arthropods have shown that AQPs are mainly involved in the physiological processes of water reabsorption, excess water excretion, anti-freezing, anti-cold, anti-drying, and other physiological processes.

The AQPs were originally speculated to play a role in the secretion of the salivary glands of *Ixodes* ticks (Sauer et al., 2000). Salivary glands are the most important organs that could absorb and excrete fluid in the body of ticks and maintain the life cycle. It not only maintains the balance of ions and water in the body but also participates in the transmission of pathogens when the tick sucks the blood of the host (Hu et al., 2020). Until present, the AQPs were identified in six tick species, including *Rhipicephalus sanguineus*, *Rhipicephalus appendiculatus*, *Rhipicephalus microplus*, *Ixodes scapularis*, *Ixodes ricinus*, and *Dermacentor variabilis* (Holmes et al., 2008; Ball et al., 2009; Guerrero et al., 2014; de Castro et al., 2016; Contreras and de la Fuente, 2017). Previous studies indicated that recombinant RmAQP1 protein was used to immunize cattle, and 3 weeks after immunization, the cattle were challenged with *R. microplus* larvae. The number of adult ticks and the total weight of ticks were significantly lower than those of the control groups. It is suggested that RmAQP1 may be used as an effective vaccine antigen to resist the challenge of *R. microplus* (Guerrero et al., 2014). These studies have validated the importance of AQPs on the physiological processes of fluid balance, such as water absorption and excretion in ticks.

At present, less is known about AQPs in *H. qinghaiensis* in China. In this study, we first described the different *AQPs* genes present in *H. qinghaiensis*. The gene characterization, protein structure of AQPs, and gene expression in different tissues and life stages were analyzed.

## Materials and Methods

### Ethical Approval

This study was approved by the Animal Ethics Committee of the Lanzhou Veterinary Research Institute, Chinese Academy of Agricultural Sciences. All animals were handled in accordance with the Animal Ethics Procedures and Guidelines on the People’s Republic of China.

### Tick Collection, Rearing, and Tissues Preparation

Adult *H. qinghaiensis* ticks were collected in spring from grass tips in Lintan County of the Gannan Tibetan Autonomous Region, and maintained in the Lanzhou Veterinary Research Institute, Chinese Academy of Agricultural Sciences. After collection, the life cycle of the tick was maintained in naïve rabbits or sheep; unfed larvae and nymphs were fed on rabbits; unfed adults were fed on sheep. After engorgement, the ticks were collected and incubated at 28°C and 80% relative humidity (RH) in glass tubes sealed with a folded filter paper and exposed to natural daylight cycle during free-living phases. The unfed adults and engorged nymphs, larvae, females, and eggs of *H. qinghaiensis* were prepared for this study. Tick tissues were dissected from partially engorged adult female ticks, according to the protocol described by Grabowski JM and Kissinger R (Grabowski and Kissinger, 2020), and the tissues were then stored in RNA later.

### Primer Design

To obtain the AQP gene sequences of *H. qinghaiensis*, a pair of degenerated primers based on two conserved motifs Asn-Pro-Ala (NPA) reported on six other tick species (i.e., *I. ricinus*, *I. scapularis*, *R. appendiculatus*, *R. sanguineus*, *R. microplus*, and *D. variabilis*; GenBank accession number: CAX48964, EEC04800, JAP83711, CAR66115, ALJ75650, and ABI53034) was designed to amplify the central region of *H. qinghaiensis* AQPs sequences. Several gene-specific primers were designed based on the partial sequences obtained and synthesized to amplify the full-length cDNA of the AQP gene using 5′ and 3′ RACE. Another pair of specific primers was designed from obtained partial 5′ and 3′ putative sequences to amplify the entire open reading frame (ORF) of the *H. qinghaiensis* AQPs gene. A pair of specific primers was designed and used to detect the quantitation of AQPs transcript present in total RNA isolated from different life cycle stages and tissues of the tick. For the standardization of the RT-qPCR, three reference genes (i.e., 18S, GAPDH, and β-actin) were initially used for normalization. But only 18S was found adequate for normalization of gene expression. We thus selected the 18S gene (GenBank accession number: MF801429) as tick reference gene. The information about all primers used in this study is shown in Table 1.

**TABLE 1:** Primers used in this study.

Primers name	Primers sequence (5′–3′)	Annealing temperature, °C
AQP-internal	F: TCCCACCTGAAYCCYGCMGTGACGCTG	55
	R: GTCTCGDGCCGGGTTSAGGGG	
5′GSP1	CCAGGTACTGCGCCACCATGTAGGGCACGA	66
5′GSP2	GACGGACGCAATGAAGGCTCCCAGGTACTG	65
5′GSP3	CCGTCGAAGTTGTCCAGGGCTCCCCTGTAT	65
5′GSP4	TCACCTACGACCGCTCGAACACC	60
5′GSP5	GGTATGAAGCGAAGATCCCCGCAGTGGC	64
5′GSP6	GGCCATTTCCGGTGCTGACGAATTCCTTGG	63
5′GSP7	GGCGAGCACCAGCAGCGCTGTGCCCACGAT	68
3′GSP8	GGTGTTCGAGCGGTCGTAGGTGA	60
3′GSP9	GCCACTGCGGGGATCTTCGCTTCATACC	64
3′GSP10	CCAAGGAATTCGTCAGCACCGGAAATGGCC	63
3′GSP11	ATCGTGGGCACAGCGCTGCTGGTGCTCGCC	68
3′GSP12	GCCATCACAGACGCCCGCAACATGGCCG	67
3′GSP13	CAGGGTGTGCAGCCCCTGTTCATTGGCTT	64
AQP-full	F: ATGTTGGAAAGCGTAAAGATCAAG	60
	R: CATCAAGACAAACGATATCAAGAATTAG	
AQP-qPCR	F:ATCACTGTCGCTGTCACCACGGT	50
	R:TTCACCTACGACCGCTCGAACA	
18S-qPCR	F:GTAGGTGAACCTGCGGAAGGAT	50
	R: GCCGCTCAGTTAGGCAAGA	

*Y: C/T, M: A/C, D: A/T/G, S: C/G.*

### gDNA and Total RNA Extraction

The ticks were first soaked in 70% ethanol for 15 min and grounded in a separate 1.5 ml tube individually to avoid cross-contamination. The tick samples were incubated with proteinase K for 2 h at 56°C and then boiled at 100°C for 10 min to inactivate proteinase K. After centrifugation, the supernatant was transferred to a fresh sterile microtube, and genomic DNA was extracted using a Genomic DNA Purification Kit (Gentra, United States) according to the instructions of the manufacturer. Total RNA from the tick materials of egg, larva, nymph, male, and female, as well as different tissues, was extracted by using a standard TRIzol reagent protocol (Life Technologies, Invitrogen, Carlsbad, CA, United States), followed by chloroform extraction, precipitation with isopropyl alcohol and ethanol, and DNase I treatment (Amplification Grade; Life Technologies, Invitrogen, Carlsbad, CA, United States). The DNA and RNA samples were stored at –20°C until further use.

### Cloning of Aquaporin From *Haemaphysalis qinghaiensis*

Degenerated primers were used to amplify a partial region of AQP from gDNA extracted from *H. qinghaiensis*, which is about 410 bp. Suitable amplification conditions were determined by gradient annealing temperature. The size of the PCR-produced amplicons was analyzed on 1.5% agarose gels with ethidium bromide staining. The crude PCR products were then cloned into TOPO TA vector (TOPO TA Cloning Kit for Sequencing; Invitrogen) and transformed into TOP10 *Escherichia coli* cells, in order to sequence the potentially different fragments. One hundred and twenty colonies were analyzed after the cloning reaction, and the size of the inserted fragment was determined by PCR using the vector primers. Plasmids were then extracted (NucleoSpin plasmid extraction; Macherey–Nagel) from the selected colonies and the insert sequence. Clones of inserts of about 410 bp were obtained, and four different sequences, all of them blasting with AQP sequences, were discovered.

Rapid amplification of 5′- and 3′-RACE-Ready cDNA from total RNA was obtained using SMARTer^®^ RACE 5′/3′ Kit (Clontech Laboratories, United States), according to the instructions of the manufacturer. The PCR products were purified and cloned into the pGEM-T easy vector (Promega, United States), followed by sequencing. The ORF was determined using ORF Finder.^1^ The full-length gene was further amplified and identified with a pair of specific primers, using cDNA and gDNA as templates.

### Bioinformatic Analysis

The sequences obtained in this study were identified using BLASTn and PSI-BLAST [non-redundant (NR) protein database] programs. A multiple sequence alignment was performed using Clustal W 2.0.12. The phylogenetic analysis was conducted using MEGA 7 software (Kumar et al., 2016). The presence of potential transmembrane domain in the *H. qinghaiensis* AQPs protein was predicted using the TMHMM Server version 2.0^2^ and topology, NetPho2.0 Server (Bustin et al., 2009).^3^ Next, the homology models of *AQPs* in *H. qinghaiensis* (*HqAQPs*) were generated with the Swiss-Model Workspace,^4^ using crystal structures of the human AQP10 (PDB ID: 6f7h) as templates.

### Detection of Gene Expression

To detect the AQP transcriptional level, the quantitative PCR was standardized to assess the gene expression of AQP in *H. qinghaiensis* in different tissues and stages. Tick stages and tissues were included: eggs, unfed larvae (approximately 50 larvae per sample), engorged nymphs (10 nymphs per sample), unfed males (10 males per sample), unfed females (10 females per sample), and individual Malpigian tubules, salivary glands, ovaries, and guts of partially engorged females (10 engorged females per sample, at day 5 of feeding). Three samples for each stage or tissue were used. The reference gene (18S) of *H. qinghaiensis* was used for normalization. RNA samples were analyzed by one-step qRT-PCR using the One Step PrimeScript RT-PCR Kit (Perfect Real Time) according to the specifications of the manufacturer (Takara, Dalian, China). Quantitative real-time PCR assays were performed on the CFX96 Touch Real-Time PCR instrument (Bio-Rad, United States) using a “quick 96-well plate.” All samples were run and analyzed in triplicate. The CFX Manager™ Software (Bio-Rad) was used to analyze the qPCR data. The transcript level of *AQP* in *H. qinghaiensis* was then calculated as a relative expression standard using the formula, which was reported by Livak and Schmittgen (2001), i.e., RNA relative expression ratio = 2**^–^**^ΔCT^, where ΔCT = (target mean Ct) – (18S mean Ct).

### Synthesis of Specific Peptides and Production of Polyclonal Antibodies

Three peptides (peptide 1: TFDKVGISGYGAAFW (37–51); peptide 2: YPKEFVSTGNGLVD (139–153); peptide 3: PARDLGPRVFTAMAG (206–220) from the *H. qinghaiensis* AQP) were synthesized by Genscript (China). Each anti-peptide-specific immune serum was prepared by immunized rabbits. Briefly, three New Zealand rabbits (2–3 kg each) were subcutaneously immunized with 500 μg of each peptide coupling to the carrier protein (KLH) *via* an N-terminal cysteine added to the peptide sequence with Freund’s complete adjuvant (FCA; Sigma, St. Louis, MO, United States; peptide: adjuvant = 1:1). Booster injections containing the same amount of each peptide in Freund’s incomplete adjuvant (FIA; Sigma) were administered on days 14 and 28. Sera were collected from the immunized rabbits before immunization or on 7 days after each immunization and interval of 20 days until 90 days and purified according to the protocol of the manufacturer (Protein A-affinity Purified, Genscript, China). The immunoglobin G (IgG) was then dialyzed against phosphate buffer saline (PBS) for 48 h, and protein concentrations were determined using Bicinchoninic Acid Assay (Pierce™ BCA Protein Assay Kit; Thermo Fisher).

### Western Blotting Analysis

The presence of *H. qinghaiensis* AQP in tick tissues and stages was assessed by Western blots. The whole proteins were extracted from eggs, unfed larvae, unfed nymphs, unfed female ticks, unfed male ticks, as well as from salivary glands, guts, Malpighian tubules, and ovaries from partially engorged female ticks (fed 5 days in sheep). The tissues or whole ticks were ground and then lysed in RIPA lysis with extraction buffers supplemented with a protease inhibitor cocktail and 1 mM Phenylmethanesulfonyl fluoride (PMSF) by rotation at room temperature (RT) for 1 h. Proteins were boiled 5 min at 100°C for denaturation, and separated by SDS-PAGE (12%) gel and subsequently transferred to nitrocellulose membranes (BioRad) at 24 V and 50 W for 35 min. The membrane was blocked with 5% (w/v) skimmed milk in tris buffer solution tween (TBST) [0.05% Tween-20 in tris buffer solution (TBS)] for 1 h at RT, washed three times in TBS (Tris–HCl, 100 mmol/L; sodium chloride, 150 mmol/L; pH 7.6) for 10 min each, and then incubated at 4°C overnight with the polyclonal antibody serving as the primary antibody, which was prepared, respectively, by immunizing rabbits with three peptides from the extracellular loops of *H. qinghaiensis* AQP. Anti-β-tubulin rabbit polyclonal antibody (Cat. no. 10094-1-AP) was used as a control. The membrane was washed three times in TBS for 10 min each, incubated in the secondary antibody (polyclonal anti-rabbit IgG–alkaline phosphatase conjugate; Sigma; A9919, dilution: 1:2,000) for 1 h at RT, and then washed three times in TBS for 10 min each. The positive blots were developed using 5-bromo-4-chloro-3-indolyphosphate (BCIP) *p*-toluidine salt/nitro-blue tetrazolium (NBT) chloride liquid substrate system (B1911-100 ml; Sigma).

### Analysis of Rabbit Antibody Response Against *Haemaphysalis qinghaiensis* Aquaporin Peptides by Enzyme-Linked Immunosorbent Assay

An indirect enzyme-linked immunosorbent assay (ELISA) was performed to detect IgG antibody against HqAQP peptide 2. Briefly, 96-well flat-bottom microplates were coated with antigen (peptide 2, 100 μl at 2 μg/ml) in coating buffer (0.1 M sodium carbonate buffer, pH 9.6) and incubated overnight at 4°C. The plates were then blocked with 5% skimmed milk in PBST for 1 h, followed by washing three times with PBS. Sera collected from rabbits diluted with 1:100 were distributed in duplicate, and the plates were incubated for 1 h at 37°C. After washing with PBS, 100 μl of anti-rabbit IgG peroxidase conjugate (Sigma, AP132P) diluted 1:15,000 was added, and the plate was incubated for 1 h at 37°C. After washing in PBS, 100 μl of 1-Step™ Ultra TMB-ELISA (34028-250 ml; Thermo Scientific) per well was added and incubated for 15 min at RT, and then stopped by adding 100 μl of 2 M sulfuric acid. The plates were read at 450 nm using an automated ELISA plate reader (Model 680 microplate reader; Bio-RAD, United States).

### Statistical Analysis

All data are presented as means ± SEM. In experiments with multiple treatments, ANOVA was performed to test for treatment effects and, if appropriate, pairwise comparisons were performed using the Tukey’s test (Minitab version 15). Two-tailed *p*-values were determined, and a *p*-value < 0.05 was considered statistically significant (*p < 0.05; ^**^p < 0.01; ^***^p < 0.001).

## Results

### *Haemaphysalis qinghaiensis* Genome Contains at Least Four Aquaporin Transcript Variants

Amplification of central region with 410 bp fragments of the AQPs gene was obtained using two degenerated primers (AQP-internal, Table 1) followed by cloning into the pGEM-T easy vector (Promega, United States) and sequence. Orthologous sequences of *R. sanguineus* (GenBank accession number: FM210537), *I. scapularis* (GenBank accession number: KT988052), and *I. ricinus* (GenBank accession number: FN178519) were identified by BLAST analysis. The sequences were found to be very similar with 10 nucleotides difference. This first result already indicated the existence of at least four distinct AQP copies in *H. qinghaiensis* genomic DNA (Supplementary Figure 1).

**FIGURE 1:**
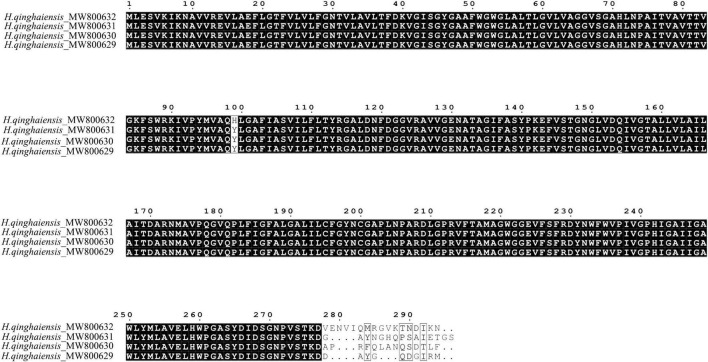
Alignment of predicted amino acid sequences of four *H. qinghaiensis* aquaporin (AQP) transcript variants.

The full-length AQP gene from *H. qinghaiensis* cDNA was amplified using 5′ and 3′ RACE. The sequence obtained was 1,702–1,723 bp and contain the predicted ORFs of 864, 876, 879, and 885 bp, a 308 bp 5′ UTR, and a 530 bp 3′ UTR following a 24 bp poly (A) tail. The full-length AQP gene was also amplified from *H. qinghaiensis* gDNA. Comparison of the sequence of the cDNA indicated that there were no introns in the AQP gene. The putative sizes of the different AQP sequences encoded the putative proteins of 287, 291, 292, and 294 amino acids, respectively. The sequences have been deposited in GenBank under the accession numbers of MW800629 (AQP1-1), MW800630 (AQP1-2), MW800631 (AQP1-3), and MW800632 (AQP1-4). Alignment of predicted amino acid sequences of four *H. qinghaiensis* AQP transcript variants indicated that the sequences are highly conserved in N-terminal and middle region, and the high polymorphic sequences occurred in C-terminal region (Figure 1).

### Sequence Comparison and Characterization of the Aquaporins Protein

**FIGURE 2:**
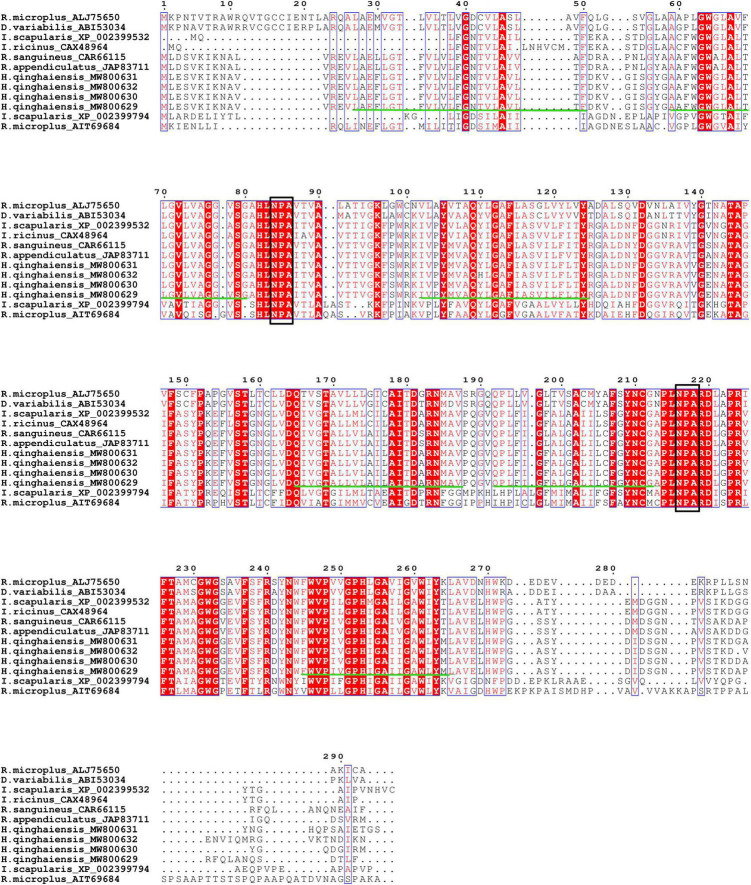
Alignment of predicted amino acid sequences of four *H. qinghaiensis* aquaporin transcript variants with reported other tick AQPs. Protein accession numbers and tick species are shown for each sequence. The two NPA motifs (asparagine-proline-alanine [Asn-Pro-Ala]) are boxed. The six transmembrane regions are indicated with green lines.

**FIGURE 3:**
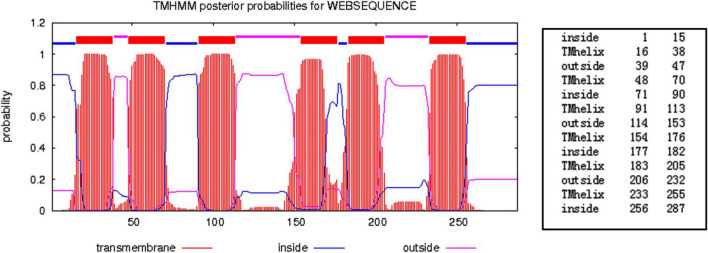
Prediction of the structure of *H. qinghaiensis* AQP topology using the TMHMM server. The values indicate the amino acids forming part of each region. Abbreviation: Tmhelix, transmembrane helix.

**FIGURE 4:**
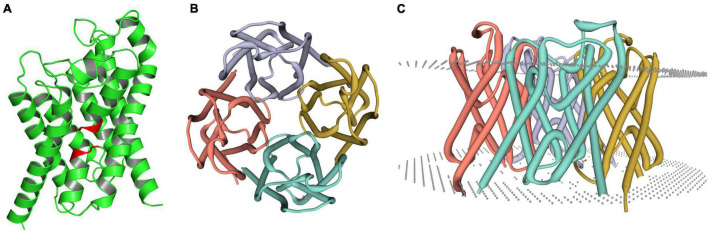
Molecular models of HqAQP1. The homology models were generated with the Swiss-Model Workspace using crystal structures of the human AQP10 (PDB ID: 6f7h) as templates. **(A)** The monomers model of HqAQP1 and the NPA sequence are labeled red color; **(B)** The tetramers model of HqAQP1; **(C)** The transmembrane model of HqAQP1. The double-layered cell membrane structure annotation is indicated by the gray dots.

Aquaporin amino acid sequences of different tick species were compared, and the results indicated that the AQP proteins of *H. qinghaiensis* have a significant identity with AQP of other tick parasites. The molecular features of the AQP family members were conserved, i.e., the 2 NPA (asparagine-proline-alanine [Asn-Pro-Ala]) motifs located at the amino acid positions 73–75 and 205–207, regions conserved in water-transporting AQPs (Figure 2). Sequence analysis using the SignalP 4.1 program indicated no signal sequence in the *HqAQP*. The program TMHMM version 2.0 predicts that *HqAQPs* had six transmembrane-spanning regions and cytosolic N- and C-termini as was standard for AQP family members (Figure 3). To investigate the structure and possible function of the predicted proteins, the homology models of HqAQP were generated with the Swiss-Model Workspace, using crystal structures of the human AQP10 (PDB ID: 6f7h) as templates. The monomers and tetramers models of HqAQP are shown in Figures 4A,B. The transmembrane model of HqAQP is shown in Figure 4C.

### Phylogenetic Analysis

**FIGURE 5:**
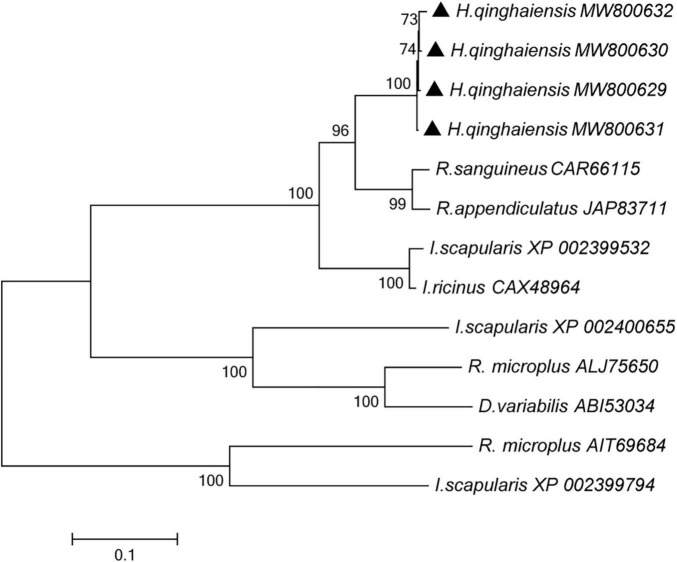
Phylogenetic tree of the amino acid sequences of *H. qinghaiensis AQP1* transcript variants and of all known members of the AQP in *R. sanguineus*, *R. appendiculatus*, *R. microplus, I. scapularis*, *I. ricinus*, and *D. variabilis.* The accession numbers were showed after parasite species name. The HqAQP1 sequences obtained in this study were indicated with bold triangle. The analysis involved 12 amino acid sequences. The tree was inferred using the neighbor-joining method of MEGA7⋅0⋅18; bootstrap values are shown at each branch point. Numbers above the branch demonstrate bootstrap support from 1,000 replications. All sites of the alignment contained insertions and deletions; missing data were eliminated from the analysis (option “complete deletion”). The optimal tree with the sum of branch length = 3.64528684. The evolutionary distances were computed using the *p*-distance method and are in the units of the number of amino acid differences per site.

A phylogenetic tree was constructed by the neighbor-joining method using the program MEGA7.0 18 based on *H. qinghaiensis* (e.g., HqAQP1-1, HqAQP1-2, HqAQP1-3, and HqAQP1-4) sequences and homologs from other related ticks deposited in GenBank, including seven species of ticks. Phylogenetic analysis of these tick AQPs suggested that they branched into two distinct clades. As expected from the similarities already described above, all the AQPs sequences of *H. qinghaiensis* formed a sister clade with the *R. sanguineus* and *R. appendiculatus* AQPs sequences in this tree (Figure 5).

### Expression of Aquaporins and Distribution in Different Tissues and Stages

The transcription level of *HqAQPs* was investigated by RT-qPCR in different tissues and life stages of *H. qinghaiensis*. The amount of transcripts of each sample was normalized to the amount of 18S expression and then calculated using the 2^–ΔCT^ method.

**FIGURE 6:**
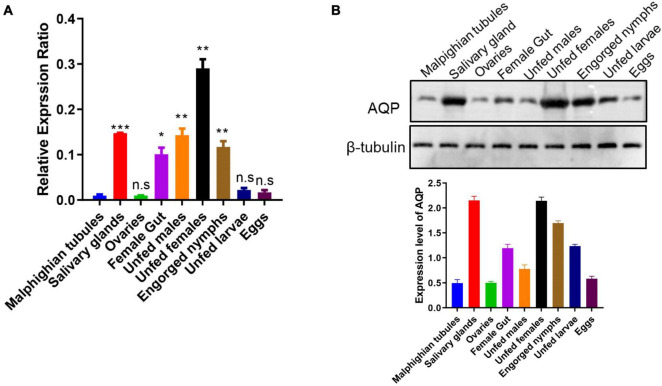
Expression of the *H. qinghaiensis* AQP (*HqAQPs*) in dissected tick tissues and different life stages. **(A)** The transcription level of *HqAQP1* was determined by RT-qPCR in Malpighian tubules, salivary glands, ovaries, female guts, unfed males, unfed females, engorged nymphs, unfed larvae, and eggs using *HqAQP1*-specific primers and was calculated as relative quantity using the delta Ct, normalized to the total amount of RNA. All experiments were independently conducted at least three times. Statistical significance is denoted by *p < 0.05, **p < 0.01, ***p < 0.001. **(B)** Western blot measurement of the pattern of expression of HqAQP1 in different tick life stages and tissues.

Relative high levels of gene expression of *HqAQPs* were found in salivary glands, female guts, as well as in unfed females, unfed males, and engorged nymphs. However, lower transcripts were detected in Malpighian tubules and ovaries of partially engorged females, as well as in eggs and unfed larvae. The relative gene expression of *HqAQP1* in the female samples was approximately 3 times higher (*p < 0.05, ^**^p < 0.01, ^***^p < 0.001) than in Malpighian tubules. Meanwhile, the relative expression in salivary glands, guts, males, and nymphs was significantly increased compared with those in Malpighian tubules (Figure 6A).

To identify native HqAQPs in *H. qinghaiensis*, antibodies from rabbits immunized with three different peptides of HqAQPs were examined for reactivity with native HqAQPs in these tick stages and tissues. The results indicated that the polyclonal antibodies from rabbits immunized with peptide 2 could recognize native AQP proteins from tick tissues and stages with different expression levels. The detected results showed that the high expression levels of HqAQPs were found in salivary glands, female guts, unfed females, and engorged nymphs, consistent with RT-qPCR results (Figure 6B).

### Analysis of Rabbit Antibody Response Against *Haemaphysalis qinghaiensis* Aquaporin Peptides by ELISA Assay

**FIGURE 7:**
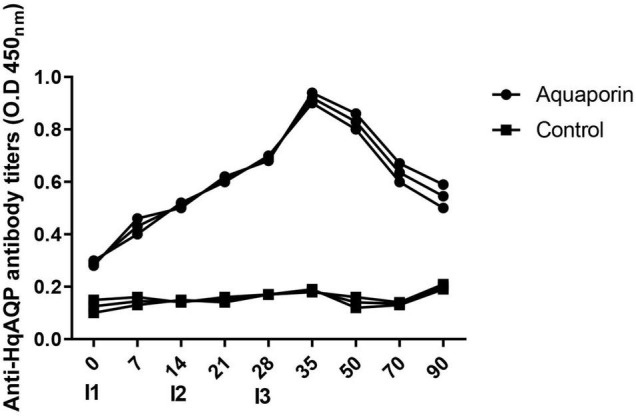
Analysis antibody response of rabbit against HqAQP1 peptide 2 by ELISA assay. Relative readings from ELISAs are plotted against trial day number for both the rabbit group and the control group inoculated with PBS. Sera was collected on days 0 (I1), 7, 14 (I2), 21, 28 (I3), 35, 50, 70, and 90 of the tests. All serum samples were analyzed in triplicate.

Furthermore, sera were used for ELISA assay from peptide 2 immunized rabbit to evaluate antibody response against HqAQP. The ELISA results showed that vaccination elicited a specific humoral immune response in inoculated rabbits. Antibody production against HqAQP was increased during the early phase of immunization at 1–4 weeks. In general, antibodies were produced after the first immunization, continued to increase after 14 days of the second immunization, and through 5 weeks, and remained stable until 12 weeks (Figure 7).

## Discussion

The geographical distribution and living habits of ticks are directly affected by global climate change (Medlock et al., 2013). New tick-borne diseases (TBDs) and recurrence of old diseases have caused global attention (Socolovschi et al., 2009; Hubálek and Rudolf, 2012; Oteo and Portillo, 2012). The control strategies mainly depend on the application of chemicals. However, intensive use of chemicals could result in resistance and environmental contamination (George et al., 2004, 2008; Graf et al., 2004; Song et al., 2014). Therefore, searching for key molecules related to fluid transport can be helpful to find an effective target for controlling the transmission of pathogens in ticks.

There are many activities of liquid transport during the transmission of pathogen and life cycle in ticks, and the fluid balance in the tick is very important to maintain its life activities. The body weight of tick females can take on up to 100 times after blood meal and concentrate the blood meal by returning approximately 75% of the ingested water and ions *via* their saliva into the host (Kaufman and Phillips, 1973).

The AQPs form homotetramer with a pore in each subunit (Holmes et al., 2008). The structures of AQPs are highly conserved among species, consisting of six transmembrane domains that are connected by two intracellular loops and three extracellular loops (Hussein et al., 2015). Two Asn-Pro-Ala (NPA) motifs are considered AQP signature motifs and are located at the protein portion that interacts to form a pore, which is the most important structural domains that play a crucial role in water-selective permeation in AQP water channels (Yakata et al., 2007; Ikeda et al., 2011). In addition to the rapid water transport across the cell membrane, the AQP may also carry small solutes, such as glycerol, urea (Liu et al., 2003), carbon dioxide (Uehlein et al., 2008), nitric oxide (Herrera et al., 2006), hydrogen peroxide (Dynowski et al., 2008), and lactic acid (Bienert et al., 2013). It is suggested that AQPs play physiologically important roles in the uptake, translocation, sequestration, or extrusion of these molecules. In hematophagous insects, AQPs have been shown to be important for regulation of water homeostasis, desiccation resistance, blood meal compaction, and general osmoregulation (Pietrantonio et al., 2000; Duchesne et al., 2003; Liu et al., 2011; Cohen, 2012). These studies demonstrate the importance of AQPs for hematophagous insects during or after blood ingestion and highlight their potential as targets for the development of novel vector control strategies.

In this study, we first reported the cloning, genetic, biological characterization and prepared the polyclonal antibodies of the AQP gene from *H. qinghaiensis*. Four different AQP transcript variants in the *H. qinghaiensis* genome resembled AQPs orthologs of other ticks sharing a similar domain organization. The AQP proteins contained all of the conserved and necessary functional motifs as described previously (Kuwahara et al., 1997). The four different AQP transcript variants found in the *H. qinghaiensis* genome are relatively well conserved, especially in six transmembrane regions, and variability is mostly limited to a 3′ region of 20 amino acids (Supplementary Figure 1 and Figure 1). These features were also reported in *R. microplus*, in which the RmAQP transmembrane helices 2–6 display more amino acid similarity than other aligned regions, and the region between predicted transmembrane helices 5 and 6 has a high number of identities in the alignment (Guerrero et al., 2014). In addition, molecular modeling suggests that HqAQP has the general AQP topology and possesses the conserved pore properties of water-specific AQPs (Shakesby et al., 2009; Goto et al., 2011; Liu et al., 2011; Philip et al., 2011; Fabrick et al., 2014).

Altogether, these results suggest that the features of *H. qinghaiensis* AQPs contain all of the motifs that are known to be conserved and necessary for function (Figures 2–4). The four putative proteins have six full transmembrane domains, two semi-transmembrane domains, and two conservative NPA (Asn-Pro-Ala) motifs, similar to the known characterizations with other tick AQPs (Holmes et al., 2008). In addition, a predicted mercury-sensitive cysteine at TMD5 suggested that the four AQPs were likely to be sensitive to mercury as demonstrated for other AQP members (Preston et al., 1993; Kuwahara et al., 1997). The phylogenetic analysis (Figure 5) maintains the relationships between the AQPs of *R. appendiculatus*, *R. sanguineus*, *R. microplus*, *I. ricinus*, and *D. variabilis* reported in previous studies with two families of AQPs noted (Ball et al., 2009; Guerrero et al., 2014). Overall, this may set the rationale for the design of novel tick killing agents targeting AQP to control *H. qinghaiensis* infestation and *H. qinghaiensis*-borne pathogens. The AQP-like protein was first reported and cloned from *D. variabilis*, which is most similar to the aquaglyceroporin AQP9 from humans. It is primarily expressed in the ovaries, which is more than 146 times compared with those in the gut. It is indicated that the tick AQP-like protein may function in the ovaries in lipid metabolism or water transport (Holmes et al., 2008).

The polyclonal antibodies were prepared, respectively, by immunizing rabbits with the synthesized peptide of the extracellular loops of *H. qinghaiensis* AQPs. AQPs from different tick tissues and stages were detected with three polyclonal antibodies. As a result, the polyclonal antibodies of anti-peptide 2 could better recognize the native antigens. According to the tetramers model of HqAQP1 (Supplementary Figure 2), we could find that the peptide 2 (blue) contains a relatively completed loop region and displays on the surface of the protein; however, the peptide 1 is located inside of the tetramer, the peptide 3 is largely located in a double helix structure, and both peptides 1 and 3 are only with a less loop region, which explains why the reactivity between antibody and protein is not better than peptide 2. Overall, it was speculated that the extracellular loop was a high confident functional region and might act as a candidate for the development of novel strategies to control *H. qinghaiensis*. So far, many biological functions of AQP in *H. qinghaiensis* are not clear, and the expression of AQPs in both organs and life stages was detected. We found that the expression of AQPs in ovaries in *H. qinghaiensis* is lower than that in the gut in both RNA and protein expression level compared with *D. variabilis* (Holmes et al., 2008).

Moreover, it has been shown in this study that HqAQP1 is found abundantly in both salivary glands and gut (Figure 6), corresponding to the tissue expression patterns in *R. sanguineus* (Ball et al., 2009). In addition, it has been found that the expression of HqAQP1 in females and nymphs is higher than other stages of the life cycle of the tick, which has less difference compared to those in *R. sanguineus*, in which the expression of RsAQP in larvae was higher than in nymphs (Ball et al., 2009). An increased expression level of HqAQP1 in these life stages may be related to a higher need of nutrients and, consequently, a need to maintain water balance through the absorption of water vapor from the air. The AQPs sequences from *H. qinghaiensis* formed a sister clade with the *R. sanguineus* and *R. appendiculatus* AQPs sequences in phylogenetic tree (Figure 5). It may have the similar expression patterns within these two tick species (Ball et al., 2009). In previous study, similar to RsAQP and HqAQP1, IrAQP of *I. ricinus* ticks is most abundantly expressed in the salivary glands of blood-sucking female ticks, followed by an expression in the intestine and gut, but not expressed in non-blood-sucking male ticks (Campbell et al., 2010). Moreover, studies have shown that knockdown of the IrAQ gene resulted in a lower blood intake and, consequently, a decrease in female tick weight (Campbell et al., 2010). Recent studies have shown that RmAQP1 from *R. microplus* has the highest expression level in the ganglia of female and male ticks, while the expression level is lower in the intestines of male ticks and ovaries of female ticks (Guerrero et al., 2014). These data suggested that the AQPs may play an important biological function in liquid transport process of both blood-sucking and non-blood-sucking phases of ticks.

Using the recombinant proteins RmAQP as antigen to immunize cattle, the result showed that both quantity and the total weight of adult ticks dropped significantly than the control group. These data indicated that RmAQP may be an effective vaccine antigen resentencing to *R. microplus* (Guerrero et al., 2014). In this study, rabbit blood was sampled weekly from each animal, and ELISA results showed that vaccination elicited a specific humoral immune response.

## Conclusion

This study first identified four AQP transcript variants and analyzed the gene expression of AQPs in different tissues, males, and females of *H. qinghaiensis*. The data presented in this study suggest that the characterization of the AQPs protein and the efficiency of peptides in the production of antibodies by mammals may be helpful for the development of new drugs and anti-tick vaccines infecting animals around China. However, even if the expression of HqAQP1 has been demonstrated in the case of *H. qinghaiensis* tick species, many studies for the detailed function of HqAQP1, together with the role of these proteins in transmitting pathogens, still remain to be further performed.

## Data Availability Statement

The datasets presented in this study can be found in online repositories. The names of the repository/repositories and accession number(s) can be found below: MW800629, MW800630, MW800631, and MW800632.

## Ethics Statement

The animal study was reviewed and approved by the Animal Ethics Committee of the Lanzhou Veterinary Research Institute, Chinese Academy of Agricultural Sciences. All animals were handled in accordance with the Animal Ethics Procedures and Guidelines on the People’s Republic of China.

## Author Contributions

QN, RH, and YP performed the experiments, including cloning, expression, Western blot analysis, and ELISA. QN and RH drafted the manuscript. ZL, JY, GG, JL, and HY supervised all work. All authors read and approved the final version of the manuscript.

## Conflict of Interest

The authors declare that the research was conducted in the absence of any commercial or financial relationships that could be construed as a potential conflict of interest.

## Publisher’s Note

All claims expressed in this article are solely those of the authors and do not necessarily represent those of their affiliated organizations, or those of the publisher, the editors and the reviewers. Any product that may be evaluated in this article, or claim that may be made by its manufacturer, is not guaranteed or endorsed by the publisher.

## Footnotes

1
www.ncbi.nlm.nih.gov/gorf

2
www.cbs.dtu.dk/services/TMHMM/

3
http://www.cbs.dtu.dk/services/NetPhos/

4
https://swissmodel.expasy.org/
