# Supplementary material for: Molecular Characterization and Gene Expression Analysis of Aquaporin in Haemaphysalis qinghaiensis
Source: Front Physiol. 2022 Feb 17;13:811628. doi: 10.3389/fphys.2022.811628 (PMC8891643; doi:10.3389/fphys.2022.811628)
Supplement: Supplementary file 2 [file Image_2.pdf]

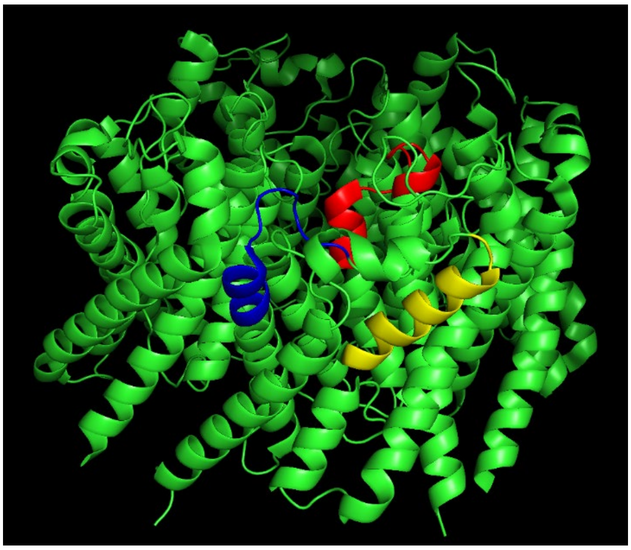

Figure S2 Localization of the three peptides in the predicted 3D models of the HqAQP1 tetramer. The homology models of HqAQPs were generated with the Swiss-Model Work-space (https://swissmodel.expasy.org/), using crystal structures of the human AQP10 (PDB ID: 6f7h) as templates. Peptide 1 is shown in red, peptide 2 is shown in blue and peptide 3 is shown in yellow.
