# Supplementary material for: Molecular Characterization and Gene Expression Analysis of Aquaporin in Haemaphysalis qinghaiensis
Source: Front Physiol. 2022 Feb 17;13:811628. doi: 10.3389/fphys.2022.811628 (PMC8891643; doi:10.3389/fphys.2022.811628)
Supplement: Supplementary file 1 [file Image_1.pdf]

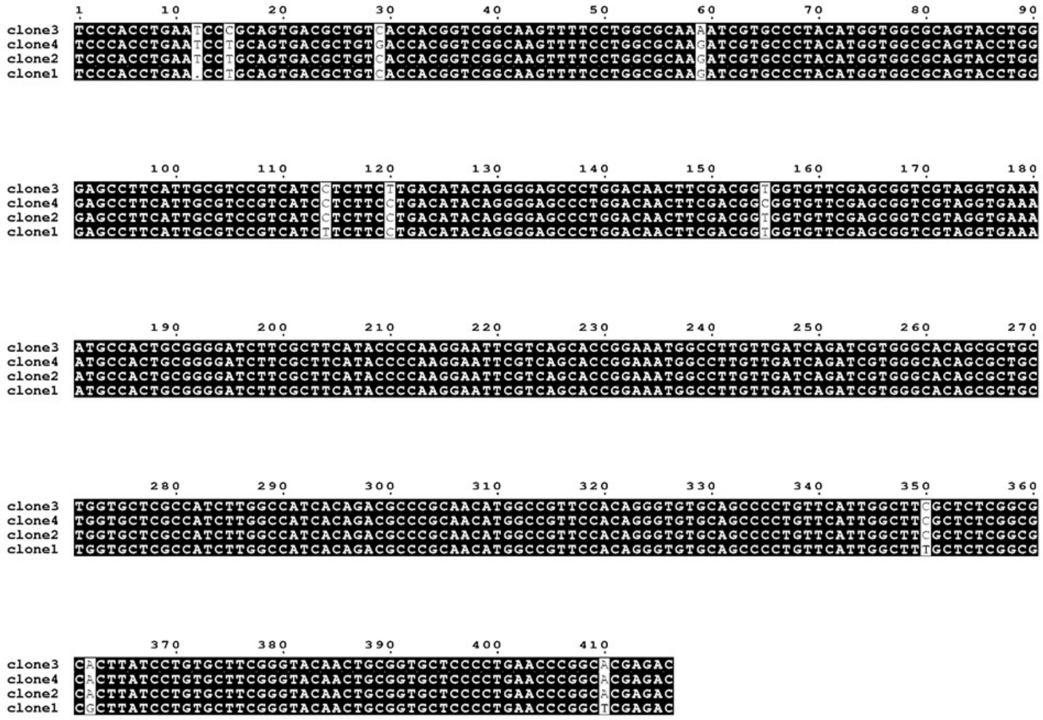

**Figure S1** Alignment of four nucleotide sequences of the aquaporin gene central regions from *H. qinghaiensis* obtained from amplification, with the degenerated primers AQP-internal, Cloning and sequencing.
